# Spectral collapse in anisotropic two-photon Rabi model

**DOI:** 10.1038/s41598-021-91915-8

**Published:** 2021-06-11

**Authors:** C. F. Lo

**Affiliations:** Institute of Theoretical Physics and Department of Physics, The Chinese University of Hong Kong, Shatin, New Territories Hong Kong

**Keywords:** Optical physics, Quantum physics

## Abstract

In this communication, based upon a squeezed-state trial wave function, we have performed a simple variational study of the spectral collapse in the anisotropic two-photon Rabi model. Our analysis indicates that the light-matter interaction and the spin-flipping (together with the anisotropy) effectively constitute two competing impacts upon the radiation mode. Whilst the former tries to decrease the radiation mode frequency, the latter may counteract or reinforce it. The light-matter interaction appears to dominate the frequency modulation as its coupling strengths go beyond the critical values, leading to the emergence of the spectral collapse. However, at the critical couplings the dominance of the light-matter interaction is not complete, and incomplete spectral collapse appears. Accordingly, at the critical couplings the eigenenergy spectrum comprises both a set of discrete energy levels and a continuous energy spectrum. The discrete eigenenergy spectrum can be derived via a simple one-to-one mapping to the bound state problem of a particle of variable effective mass in a finite potential well, and the number of bound states available is determined by the energy difference between the two atomic levels. Each of these eigenenergies has a twofold degeneracy corresponding to the spin degree of freedom.

## Introduction

In recent years the quantum two-photon Rabi model has been attracting much attention in the literature because it exhibits a counter-intuitive feature, commonly known as the “spectral collapse” , which occurs when the light-matter coupling strength $\epsilon$ goes beyond a critical value $\epsilon _{c}$^1–11^ . The spectral collapse was first demonstrated by Ng et al. in 1998^12,13^ by means of exact numerical diagonalization, and was shown to be generated by a fundamental change of the system due to the intensification of the light-matter interaction. As pointed out by the authors, in the special case of vanishing energy difference $\omega _{0}$ between the two atomic levels, the system behaves as a simple harmonic oscillator for $\epsilon <\epsilon _{c}$ and as an inverted harmonic potential barrier for $\epsilon >\epsilon _{c}$. Thus, in the subcritical regime the system has a discrete set of eigenenergies whereas the discrete energy spectrum turns into a continuous one as $\epsilon$ goes beyond the critical value $\epsilon _{c}$. In addition, such a structural change of the eigenenergy spectrum persists for $\omega _{0}\ne 0$ because this spin-flip term is a bounded operator. Since then, a number of theoretical studies on the two-photon Rabi model have confirmed the results and observations of Ng et al., via both analytical and numerical investigations^14–23^ . Recently, via an elementary quantum mechanics approach, Lo^24^ has also demonstrated explicitly that at the critical coupling the eigenenergy spectrum consists of both a set of discrete energy levels and a continuous energy spectrum, indicating an incomplete spectral collapse at the critical coupling. Moreover, recent advancement in the state-of-the-art quantum technology has dramatically made the applications of the two-photon Rabi model feasible in the strong coupling regime^1,2,4,11^.

Stimulated by such a success, some people have started to pay special attention to some generalizations of the two-photon Rabi model. The simplest generalization is the anisotropic two-photon Rabi model, in which the rotating and counter-rotating terms have different coupling parameters. By means of a variation of Braak’s G-function method based upon Bogoliubov rotation of the underlying $su\left( 1,1\right)$ Lie algebra^25^, Cui et al.^26^ have performed an exact analysis of its spectral properties and found condensation of discrete energy levels, i.e. spectral collapse, when the critical values of light-matter couplings are breached. However, it should be noted that the G-function analysis can only approach the critical coupling limits asymptotically. It is thus the aim of this paper to scrutinize the spectral collapse in the anisotropic two-photon Rabi model, particularly the characteristic behaviour of the eigenenergy spectrum at the critical couplings.

The structure of this paper is organized as follows. In “Squeezed-state trial wave function” section a simple variational study based upon a squeezed-state trial wave function is performed to demonstrate rigorously how the spectral collapse in the anisotropic two-photon Rabi model occurs as its coupling strengths go beyond the critical values. A simple physical picture providing intuitive insights for the underlying physics is presented. In “Incomplete spectral collapse” section the eigenenergy spectrum of the system at the critical couplings is investigated. The system has an incomplete spectral collapse, implying that the eigenenergy spectrum comprises both a set of discrete energy levels and a continuous energy spectrum. The discrete eigenenergy spectrum can be derived via a simple one-to-one mapping to the bound state problem of a particle of variable effective mass in a finite potential well. The depth of the potential well is proportional to the energy difference between the two atomic levels so that the extent of the spectral collapse (or the number of bound states available) can be monitored straightforwardly. The final section conludes the paper.

## Squeezed-state trial wave function

As in Cui et al.^26^, the Hamiltonian of the anisotrpic two-photon Rabi model is given by $\left( \hbar =1\right)$

1 $$\begin{aligned} H=& {} \omega a^{\dag }a+\Delta \sigma _{z}+g\left( \sigma _{+}a^{2}+\sigma _{-}a^{\dag 2}\right) +\lambda g\left( \sigma _{+}a^{\dag 2}+\sigma _{-}a^{2}\right) \nonumber \\= & {} \omega a^{\dag }a+\omega _{0}S_{z}+2g_{1}\left( a^{\dag 2}+a^{2}\right) S_{x}+i2g_{2}\left( a^{2}-a^{\dag 2}\right) S_{y}, \end{aligned}$$

for $\sigma _{\pm }=\frac{1}{2}\left( \sigma _{x}\pm i\sigma _{y}\right)$, $\vec {S}=\frac{1}{2}\vec {\sigma }$, $\omega _{0}=2\Delta$, $g_{1}=\frac{1}{2} g\left( 1+\lambda \right)$ and $g_{2}=\frac{1}{2}g\left( 1-\lambda \right)$ . Here the radiation mode of frequency $\omega$ is described by the bosonic operators $\left\{ a,a^{\dag }\right\}$, the two atomic levels separated by an energy difference $\omega _{0}$ are represented by the spin-half operators $\left\{ S_{x},S_{y},S_{z}\right\}$, the light-matter coupling strength is measured by the positive parameter *g*, and $\lambda$ is the anisotropy parameter. Without loss of generality, we may assume that both $g_{1}$ and $g_{2}$ are non-negative definite, i.e. $-1\le \lambda \le 1$. In addition, under the unitary transformation $U=\exp \left\{ i\frac{1}{2}\pi S_{z}\right\} \exp \left\{ i\frac{1}{4}\pi a^{\dag }a\right\}$, the Hamiltonian *H* becomes

2 $$\begin{aligned} \mathcal{H}=U^{\dag }HU=\omega a^{\dag }a+\omega _{0}S_{z}+2g_{2}\left( a^{\dag 2}+a^{2}\right) S_{x}+i2g_{1}\left( a^{2}-a^{\dag 2}\right) S_{y}. \end{aligned}$$

Evidently, $\mathcal{H}$ bears a striking resemblance to *H*, except that $g_{1}$ and $g_{2}$ are interchanged. In the following we shall investigate the critical values of the coupling parameters $g_{1}$ and $g_{2}$. For simplicity, we set the energy unit in such a way that $\omega =1$.

To begin with, we try to examine the critical value of $g_{1}$ under the assumption that $g_{2}<g_{1}$. By applying the unitary transformation

3 $$\begin{aligned} R=\exp \left\{ -\frac{i\pi }{2}\left( S_{z}-\frac{1}{2}\right) \right\} a^{\dag }a \end{aligned}$$

to the Hamiltonian *H*, we obtain

4 $$\begin{aligned} {\tilde{H}} & = R^{\dag }HR \nonumber \\ & = \left[ \omega _{0}-2g_{2}\left( a^{2}-a^{\dag 2}\right) \right] \left\{ \cos \left( \frac{\pi }{2}a^{\dag }a\right) S_{z}+\sin \left( \frac{\pi }{2} a^{\dag }a\right) S_{y}\right\} +a^{\dag }a \nonumber \\ & \quad + g_{1}\left( a^{\dag 2}+a^{2}\right) . \end{aligned}$$

It is not difficult to realize that within the subspace of even number states of $a^{\dag }a$ the transformed Hamiltonian ${\tilde{H}}$ becomes

5 $$\begin{aligned} {\tilde{H}}_{e}=\left[ \omega _{0}-2g_{2}\left( a^{2}-a^{\dag 2}\right) \right] \cos \left( \frac{\pi }{2}a^{\dag }a\right) S_{z}+a^{\dag }a+g_{1}\left( a^{\dag 2}+a^{2}\right), \end{aligned}$$

whereas within the subspace of odd number states we have

6 $$\begin{aligned} {\tilde{H}}_{o}=\left[ \omega _{0}-2g_{2}\left( a^{2}-a^{\dag 2}\right) \right] \sin \left( \frac{\pi }{2}a^{\dag }a\right) S_{y}+a^{\dag }a+g_{1}\left( a^{\dag 2}+a^{2}\right) . \end{aligned}$$

In both cases the spin degree of freedom and the boson mode are decoupled. Thus, the eigenstates of each subspace are simply given by the product states $\left| M\right\rangle \left| \phi _{n}\right\rangle$, where $\left| M\right\rangle$ is an eigenstate of the spin operator $S_{z}$ or $S_{y}$, and $\left| \phi _{n}\right\rangle$ the *n*-th eigenstate of the one-body bosonic Hamiltonian

7 $$\begin{aligned} {\bar{H}}=M\left[ \omega _{0}-2g_{2}\left( a^{2}-a^{\dag 2}\right) \right] F\left( a^{\dag }a\right) +a^{\dag }a+g_{1}\left( a^{\dag 2}+a^{2}\right), \end{aligned}$$

where $$M=\pm \frac{1}{2}$$ and

8 $$\begin{aligned} F\left( a^{\dag }a\right) =\left\{ \begin{array}{cc} \cos \left( \frac{\pi }{2}a^{\dag }a\right) &{} \text {for the subspace of even number states} \\ \sin \left( \frac{\pi }{2}a^{\dag }a\right) &{} \text {for the subspace of odd number states} \end{array} \right. . \end{aligned}$$

As a result, the Hilbert space of *H* is divided into four different subspaces, each of which is specified by the spin quantum number and the parity.

Likewise, in the special case of $\omega _{0}=g_{2}=0$ the one-body Hamiltonian ${\bar{H}}$ can be diagonalized by the unitary squeezing transformation $T=\exp \left\{ -\frac{1}{4}\tanh ^{-1}\left( 2g_{1}\right) \left( a^{\dag 2}-a^{2}\right) \right\}$ for $g_{1}<\frac{1}{2}$ as follows^12^:

9 $$\begin{aligned} \mathcal{H}=T^{\dagger }{\bar{H}}T={\tilde{\omega }}\left( a^{\dag }a+\frac{1}{2} \right) -\frac{1}{2}\ , \end{aligned}$$

where ${\tilde{\omega }}=\sqrt{1-4g_{1}^{2}}<1$, implying that the transformed radiation mode has a lower frequency, and that the light-matter interaction has the effect of generating a redshift to the radiation mode frequency. On the other hand, there is no unitary transformation which can diagonalize ${\bar{H}}$ for $g_{1}>\frac{1}{2}$. Indeed, as pointed out by Ng et al.^12,13^, the system represents a simple harmonic oscillator for $g_{1}<\frac{1}{2}$, becomes a free particle at the critical coupling, *i*.*e*. $g_{1}=\frac{1}{2}$, and finally turns into an inverted harmonic potential barrier for $g_{1}>\frac{1}{2}$. Such an abrupt structural change of the system thus leads to the collapse of a set of discrete eigenenergy levels into a continuum energy spectrum in this special case.

Now we perform a variational study of the ground state in each of the four subspaces. In view of the aforementioned unitary squeezing transformation, a natural candidate for the variational wave function is the squeezed-state trial wave function:

10 $$\begin{aligned} |G\rangle =\exp \left\{ \frac{1}{4}\xi \left( a^{\dag 2}-a^{2}\right) \right\} |\Psi \rangle \end{aligned}$$

for some real variational parameter $\xi$, where

11 $$\begin{aligned} |\Psi \rangle =\left\{ \begin{array}{cc} |0\rangle &{} \text {for the subspace of even number states} \\ |1\rangle &{} \text {for the subspace of odd number states} \end{array} \right. . \end{aligned}$$

With respect to this trial wave function, the expectation value of ${\bar{H}}$ can be calculated readily:

12 $$\begin{aligned} E&= \langle G|{\bar{H}}|G\rangle \nonumber \\&= M\omega _{0}\text {sech}^{2k}\left( \xi \right) \left\{ 1+\frac{8kg_{2}}{ \omega _{0}}\tanh \left( \xi \right) \right\} +2k\left[ \cosh \left( \xi \right) +2g_{1}\sinh \left( \xi \right) \right] -\frac{1}{2} \nonumber \\&= M\omega _{0}\text {sech}^{2k}\left( \xi \right) \left\{ 1+\frac{8kg_{2}}{ \omega _{0}}\tanh \left( \xi \right) \right\} +k\left[ \left( 1+2g_{1}\right) e^{\xi }+\left( 1-2g_{1}\right) e^{-\xi }\right] -\frac{1}{2},\ \end{aligned}$$

where

13 $$\begin{aligned} k=\left\{ \begin{array}{cc} \frac{1}{4} &{} \text {for the subspace of even number states} \\ \frac{3}{4} &{} \text {for the subspace of odd number states} \end{array} \right. . \end{aligned}$$

Apparently, for $g_{1}<\frac{1}{2}$ the second term of *E*, *i.e*. the term with the square brackets, is positive definite for all values of $\xi$ so that *E* approaches infinity as $\xi \rightarrow \pm \infty$ for

$$\begin{aligned} \left| M\omega _{0}\text {sech}^{2k}\left( \xi \right) \left\{ 1+\frac{ 8kg_{2}}{\omega _{0}}\tanh \left( \xi \right) \right\} \right| <\frac{ \omega _{0}}{2}\left( 1+\frac{8kg_{2}}{\omega _{0}}\right), \end{aligned}$$

regardless of *k*. Thus, a minimum value of *E* is guaranteed for each subspace. For $\omega _{0}=g_{2}=0$, the minimum appears at $\xi =$ $-\tanh ^{-1}\left( 2g_{1}\right) \equiv \xi _{0}$, giving the exact energy $E=2{\tilde{\omega }}k-\frac{1}{2}$ for ${\tilde{\omega }}=\sqrt{1-4g_{1}^{2}}$^12,13^. It is obvious that the light-matter interaction tries to decrease the radiatioon mode frequency. Then, once the spin-flip term is turned on, the minimum moves above $\xi _{0}$ for $M=-\frac{1}{2}$ and below $\xi _{0}$ for $M=\frac{1}{2}$, regardless of *k*. That is, the spin-flipping counteracts the light-matter interaction for $M=-\frac{1}{2}$ whilst reinforcing it for $M=\frac{1}{2}$. Similar observations can be made for a finite $g_{2}$. On the other hand, for $g_{1}>\frac{1}{2}$ the term with the square brackets decreases monotonically as $\xi$ approaches $-\infty$, indicating that *E* is not bounded below. This is in agreement with the numerical diagonalization of ${\bar{H}}$ using the basis states in each subspace, which does not give any converged results at all, indicating the non-existence of bound states.

Finally, we examine the critical value of $g_{2}$ under the assumption that $g_{1}<g_{2}$. Since $\mathcal{H}$ closely resembles *H*, except that $g_{1}$ and $g_{2}$ are interchanged, the aforementioned analysis can be straightforwardly applied to $\mathcal{H}$. Beyond question, the same conclusion is reached; for $g_{2}<\frac{1}{2}$ a normalisable ground state exists in each subspace whilst for $g_{2}>\frac{1}{2}$ there is no bound state at all. Accordingly, the critical values of $g_{1}$ and $g_{2}$ are both equal to $\frac{1}{2}$, implying that a discrete eigenenergy spectrum exists for the confined region specified by $g_{1}<\frac{1}{2}$ and $g_{2}< \frac{1}{2}$ only.

## Incomplete spectral collapse

In this section we apply Lo’s approach^24^ to determine the eigenstates of the one-body Hamiltonian ${\bar{H}}$ in both subspaces at the critical couplings. First of all, we examine the case that $g_{2}<g_{1}$ and $g_{1}=\frac{1}{2}$. In terms of the “position” and “momentum” operators of the boson mode:

14 $$\begin{aligned} x=\frac{1}{\sqrt{2}}\left( a+a^{\dag }\right) \qquad \text {and}\qquad p= \frac{1}{i\sqrt{2}}\left( a-a^{\dag }\right), \end{aligned}$$

we rewrite ${\bar{H}}$ as

15 $$\begin{aligned} {\bar{H}}=i^{\nu }\sqrt{i}M\omega _{0}\left[ 1-i\frac{2g_{2}}{\omega _{0}} \left( xp+px\right) \right] \exp \left( -\frac{i\pi }{2}H_{0}\right) +\left( H_{0}-\frac{1}{2}\right) -\frac{1}{2}\left( p^{2}-x^{2}\right), \end{aligned}$$

where

16 $$\begin{aligned} H_{0}=\frac{p^{2}}{2}+\frac{x^{2}}{2}=a^{\dag }a+\frac{1}{2} \end{aligned}$$

is the Hamiltonian of a quantum simple harmonic oscillator of unit mass and frequency, and

17 $$\begin{aligned} \nu =\left\{ \begin{array}{cc} 0 &{} \text {for the subspace of even number states} \\ 1 &{} \text {for the subspace of odd number states} \end{array} \right. . \end{aligned}$$

In the coordinate space the eigenvalue equation of ${\bar{H}}$ reads

18 $$\begin{aligned} E\phi \left( x\right)&=\left\{ i^{\nu }\sqrt{i}M\omega _{0}\left[ 1-i\frac{ 2g_{2}}{\omega _{0}}\left( xp+px\right) \right] \exp \left( -\frac{i\pi }{2} H_{0}\right) \phi \left( x\right) +x^{2}-\frac{1}{2}\right\} \phi \left( x\right) \nonumber \\&= \left( x^{2}-\frac{1}{2}\right) \phi \left( x\right) +i^{\nu }M\omega _{0} \left[ 1-\frac{2g_{2}}{\omega _{0}}\left( x\frac{d}{dx}+\frac{d}{dx}x\right) \right] {\tilde{\phi }}\left( x\right), \end{aligned}$$

where *E* denotes the eigenenergy and

19 $$\begin{aligned} {\tilde{\phi }}\left( p\right) =\int _{-\infty }^{\infty }\frac{dx}{\sqrt{2\pi }} \exp \left\{ -ipx\right\} \phi \left( x\right) \end{aligned}$$

is the Fourier transform of $\phi \left( x\right)$. Here we have made use of the fact that

20 $$\begin{aligned} \exp \left( -itH_{0}\right) \phi \left( x\right) =\int _{-\infty }^{\infty }dyK\left( x,t;y\right) \phi \left( y\right), \end{aligned}$$

where $K\left( x,t;y\right)$ is the propagator of $H_{0}$ defined by

21 $$\begin{aligned} K\left( x,t;y\right) =\frac{1}{\sqrt{i2\pi \sin \left( t\right) }}\exp \left\{ -\frac{\left( x^{2}+y^{2}\right) \cos \left( t\right) -2xy}{i2\sin \left( t\right) }\right\} . \end{aligned}$$

Similarly, in the momentum space the eigenvalue equation of ${\bar{H}}$ is given by

22 $$\begin{aligned} E{\tilde{\phi }}\left( p\right) =\left( -\frac{d^{2}}{dp^{2}}-\frac{1}{2} \right) {\tilde{\phi }}\left( p\right) +\left( -i\right) ^{\nu }M\omega _{0} \left[ 1+\frac{2g_{2}}{\omega _{0}}\left( p\frac{d}{dp}+\frac{d}{dp}p\right) \right] \phi \left( p\right), \end{aligned}$$

Eliminating $\phi \left( x\right)$ from Eqs. (18) and (22) yields

23 $$\begin{aligned} \left( E+\frac{1}{2}\right) {\tilde{\phi }}\left( p\right) & = {} -\frac{d^{2} {\tilde{\phi }}\left( p\right) }{dp^{2}}+\frac{1}{4}\left\{ \omega _{0}+2g_{2}\left( p\frac{d}{dp}+\frac{d}{dp}p\right) \right\} \frac{1}{E+ \frac{1}{2}-p^{2}} \nonumber \\ & \quad \times\left\{ \omega _{0}-2g_{2}\left( p\frac{d}{dp}+\frac{d}{dp}p\right) \right\} {\tilde{\phi }}\left( p\right), \end{aligned}$$

for $M^{2}=1/4$ and $i^{\nu }\left( -i\right) ^{\nu }=1$. The disappearance of both the parameter $\nu$ and spin eigenvalue *M* in Eq. (23) implies that the even-parity and odd-parity eigenstates of $\tilde{H }$ simply correspond to the even-parity and odd-parity solutions of the eigenvalue equation, respectively, and that each eigenstate is doubly degenerate. For $E+\frac{1}{2}<0$, we introduce the parameter $\kappa = \sqrt{\left| E+\frac{1}{2}\right| }$ and define a new variable $q=p/\kappa$ such that Eq. (23) can be expressed as

24 $$\begin{aligned} -\kappa ^{4}{\tilde{\phi }}\left( q\right) & = -\frac{d^{2}{\tilde{\phi }}\left( q\right) }{dq^{2}}-\left\{ \frac{\omega _{0}}{2}+g_{2}\left( q\frac{d}{dq}+ \frac{d}{dq}q\right) \right\} \frac{1}{1+q^{2}} \nonumber \\ & \quad \times \left\{ \frac{\omega _{0}}{2}-g_{2}\left( q\frac{d}{dq}+\frac{d}{dq} q\right) \right\} {\tilde{\phi }}\left( q\right) \nonumber \\ & = \left\{ -\frac{d}{dq}\left[ \frac{1}{2m\left( q\right) }\right] \frac{d}{ dq}+V\left( q\right) \right\} {\tilde{\phi }}\left( q\right), \end{aligned}$$

where

25 $$\begin{aligned} m\left( q\right)= & {} \frac{1+q^{2}}{2\left[ 1+\left( 1-4g_{2}^{2}\right) q^{2} \right] } \end{aligned}$$

26 $$\begin{aligned} V\left( q\right)= & {} -\frac{\left( \omega _{0}/2\right) ^{2}-g_{2}^{2}}{ \left( 1+q^{2}\right) ^{2}}\left\{ 1+\frac{\left( \omega _{0}/2\right) -3g_{2}}{\left( \omega _{0}/2\right) +g_{2}}q^{2}\right\} . \end{aligned}$$

Accordingly, we have obtained the time-independent Schrödinger equation for a particle of variable effective mass $m\left( q\right)$ in the finite potential well $V\left( q\right)$^27,28^. Since $g_{2}< \frac{1}{2}$ in this case, the variable effective mass $m\left( q\right)$ is positive definite. Here $V\left( q\right)$ describes a simple finite potential well for $g_{2}<\frac{1}{2}\omega _{0}$ whilst it behaves like a finite double-well potential for $g_{2}>\frac{1}{2}\omega _{0}$. It should be noted that in the absence of anisotropy, *i*.*e*. $g_{2}=0$, the variable effective mass $m\left( q\right)$ is reduced to a constant and the finite potential well $V\left( q\right)$ becomes a “Lorentzian function” potential well^24^. Likewise, as shown in Lo^29^, we may perform a suitable unitary transformation to reduce Eq. (24) to the time-independent Schrödinger equation for a particle of unit mass in a finite potential well (see “Appendix”).

On the other hand, for $E+\frac{1}{2}>0$, in terms of the parameter $k=\sqrt{ E+\frac{1}{2}}$ and the new variable ${\bar{q}}=p/k$, Eq. (23) becomes

27 $$\begin{aligned} k^{4}{\tilde{\phi }}\left( {\bar{q}}\right) =\left\{ -\frac{d}{d{\bar{q}}}\left[ \frac{1}{2{\bar{m}}\left( {\bar{q}}\right) }\right] \frac{d}{d{\bar{q}}}+{\bar{V}} \left( {\bar{q}}\right) \right\} {\tilde{\phi }}\left( {\bar{q}}\right), \end{aligned}$$

where

28 $$\begin{aligned} {\bar{m}}\left( {\bar{q}}\right)= & {} \frac{1-{\bar{q}}^{2}}{2\left[ 1-\left( 1-4g_{2}^{2}\right) {\bar{q}}^{2}\right] } \end{aligned}$$

29 $$\begin{aligned} {\bar{V}}\left( {\bar{q}}\right)= & {} \frac{\left( \omega _{0}/2\right) ^{2}-g_{2}^{2}}{\left( 1-{\bar{q}}^{2}\right) ^{2}}\left\{ 1-\frac{\left( \omega _{0}/2\right) -3g_{2}}{\left( \omega _{0}/2\right) +g_{2}}{\bar{q}} ^{2}\right\} . \end{aligned}$$

Obviously, this is the time-independent Schrödinger equation of the scattering state problem associated with a particle of variable effective mass ${\bar{m}}\left( {\bar{q}}\right)$ in the presence of the potential barrier ${\bar{V}}\left( {\bar{q}}\right)$ that is singular at ${\bar{q}}=\pm 1$.

Finally, the aforementioned analysis can be readily applied to the case that $g_{1}<g_{2}$ and $g_{2}=\frac{1}{2}$ by simply replacing $g_{2}$ by $g_{1}$ in Eq. (23) and the subsequent equations because $\mathcal{H}$ can be derived from *H* by interchanging $g_{1}$ and $g_{2}$. Beyond question, the same conclusion can be drawn.

## Conclusion

Based upon a squeezed-state trial wave function, we have performed a simple variational study of the spectral collapse in the anisotropic two-photon Rabi model. Our analysis indicates that the light-matter interaction and the spin-flipping (together with the anisotropy) effectively constitute two competing impacts upon the radiation mode. Whilst the former tries to decrease the radiation mode frequency, the latter may counteract or reinforce it. The light-matter interaction appears to dominate the frequency modulation as its coupling strengths go beyond the critical values, namely $g_{1}>\frac{1}{2}$ and/or $g_{2}>\frac{1}{2}$, leading to the emergence of the spectral collapse. However, at the critical couplings the dominance of the light-matter interaction is not complete, and incomplete spectral collapse appears. Accordingly, at the critical couplings the eigenenergy spectrum comprises both a set of discrete energy levels and a continuous energy spectrum. The discrete eigenenergy spectrum can be derived via a simple one-to-one mapping to the bound state problem of a particle of variable effective mass in a finite potential well, and the number of bound states available is determined by the energy difference between the two atomic levels. Each of these eigenenergies has a twofold degeneracy corresponding to the spin degree of freedom.

## Appendix

In order to faciliate a better understanding of the eigenenergy spectrum, we introduce the unitary transformation^29^

30 $$\begin{aligned} U=\exp \left\{ \frac{i}{2}\left[ \left( \frac{1}{i}\frac{d}{dq}\right) f\left( q\right) +f\left( q\right) \left( \frac{1}{i}\frac{d}{dq}\right) \right] \right\} \end{aligned}$$

for some function $f\left( q\right)$, which transforms *q* and $\frac{1}{i} \frac{d}{dq}$ as follows:

31 $$\begin{aligned} U^{\dag }qU= & {} q+F\left( q\right) \end{aligned}$$

32 $$\begin{aligned} U^{\dag }\left( \frac{1}{i}\frac{d}{dq}\right) U= & {} \frac{1}{2}\left\{ \left( \frac{1}{i}\frac{d}{dq}\right) G\left( q\right) +G\left( q\right) \left( \frac{1}{i}\frac{d}{dq}\right) \right\} . \end{aligned}$$

Here

33 $$\begin{aligned} F\left( q\right)= & {} \sum _{n=1}^{\infty }\frac{\left( -1\right) ^{n}}{n!} f_{n}\left( q\right),\quad f_{n+1}\left( q\right) =f\left( q\right) \frac{df_{n}\left( q\right) }{dq},\quad f_{1}\left( q\right) =f\left( q\right) \end{aligned}$$

34 $$\begin{aligned} G\left( q\right)= & {} \sum _{n=0}^{\infty }\frac{\left( -1\right) ^{n}}{n!} g_{n}\left( q\right),\quad g_{n+1}\left( q\right) =f^{2}\left( q\right) \frac{d}{dq}\left\{ \frac{g_{n}\left( q\right) }{f\left( q\right) } \right\},\quad g_{0}\left( q\right) =1. \end{aligned}$$

Obviously, in order that the commutation relation between *q* and $\frac{1}{i }\frac{d}{dq}$ are preserved under the unitary transformation *U*, *i*. *e*. $\left[ U^{\dag }qU,U^{\dag }\left( \frac{1}{i}\frac{d}{dq}\right) U \right] =\left[ q,\frac{1}{i}\frac{d}{dq}\right] =i$, we must require

35 $$\begin{aligned} \frac{d}{dq}\left\{ q+F\left( q\right) \right\} =\frac{1}{G\left( q\right) } . \end{aligned}$$

Then, applying the unitary transformation *U* to Eq. (24) gives

36 $$\begin{aligned} -\kappa ^{4}{\tilde{\phi }}\left( q\right) & = \left\{ -\frac{d}{dq}\left( \frac{G\left( q\right) ^{2}}{2m\left( \xi \left( q\right) \right) }\right) \frac{d}{dq}-\frac{1}{8}\left[ \frac{d}{dq},\frac{2G\left( q\right) }{ m\left( \xi \left( q\right) \right) }\frac{dG\left( q\right) }{dq}\right] \right. \nonumber \\ & \quad \left. + \frac{1}{8m\left( \xi \left( q\right) \right) }\left( \frac{ dG\left( q\right) }{dq}\right) ^{2}+V\left( m\left( \xi \left( q\right) \right) \right) \right\} {\tilde{\phi }}\left( q\right), \end{aligned}$$

where $\xi \left( q\right) =q+F\left( q\right)$ and ${\bar{\phi }}\left( q\right) =U^{\dag }\phi \left( q\right)$. By setting $G\left( q\right) = \sqrt{m\left( \xi \left( q\right) \right) }$, we obtain

37 $$\begin{aligned} \left[ \frac{d}{dq},\frac{2G\left( q\right) }{m\left( \xi \left( q\right) \right) }\frac{dG\left( q\right) }{dq}\right] =\frac{d}{dq}\left\{ \frac{ 2G\left( q\right) }{m\left( \xi \left( q\right) \right) }\frac{dG\left( q\right) }{dq}\right\} =\frac{d^{2}\ln m\left( \xi \left( q\right) \right) }{ dq^{2}} \end{aligned}$$

and

38 $$\begin{aligned} \frac{1}{m\left( \xi \left( q\right) \right) }\left( \frac{dG\left( q\right) }{dq}\right) ^{2}=\frac{1}{4}\left\{ \frac{d\ln m\left( \xi \left( q\right) \right) }{dq}\right\} ^{2}. \end{aligned}$$

As a result, Eq. (36) is reduced to

39 $$\begin{aligned} -\kappa ^{4}{\tilde{\phi }}\left( q\right) =\left\{ -\frac{1}{2}\frac{d^{2}}{ dq^{2}}+{\tilde{V}}\left( \xi \left( q\right) \right) \right\} {\tilde{\phi }} \left( q\right), \end{aligned}$$

where

40 $$\begin{aligned} {\bar{V}}\left( \xi \left( q\right) \right)= & {} V\left( \xi \left( q\right) \right) -\frac{1}{8}\left( \frac{d^{2}\ln m\left( \xi \left( q\right) \right) }{dq^{2}}\right) +\frac{1}{32}\left( \frac{d\ln m\left( \left( q\right) \right) }{dq}\right) ^{2} \nonumber \\= & {} V\left( \xi \left( q\right) \right) -\frac{4g_{2}^{2}}{\left( 1+\xi \left( q\right) ^{2}\right) ^{3}}\left\{ \frac{1-g_{2}^{2}\xi \left( q\right) ^{2}}{1+\left( 1-4g_{2}^{2}\right) \xi \left( q\right) ^{2}}-\frac{3 }{2}\xi \left( q\right) ^{2}\right\} . \end{aligned}$$

By taking a close look at the ${\bar{V}}\left( \xi \right)$, we can readily recognise that it represents a symmetric finite potential well in $\xi$ with its minimum value occurring at $\xi =0$: ${\bar{V}}\left( \xi =0\right) =-\left\{ \left( \omega _{0}/2\right) ^{2}+3g_{2}^{2}\right\}$, and it vanishes asymptotically as $\xi \rightarrow \pm \infty$. In addition, Eq. (35) yields

41 $$\begin{aligned} q\left( \xi \right) =\int _{0}^{\xi }d\eta \sqrt{m\left( \eta \right) }\ , \end{aligned}$$

indicating that *q* is a monotonically increasing function of $\xi$. Consequently, we have succeeded in transforming Eq. (24) into the time-independent Schrödinger equation of the bound state problem associated with a particle of unit mass moving in the finite potential well ${\bar{V}}\left( \xi \left( q\right) \right)$, and this system has a set of discrete bound state eigenenergy levels.

## References

[1] Felicetti S (2015). Spectral collapse via two-photon interactions in trapped ions. Phys. Rev. A.

[2] Puebla R, Hwang MJ, Casanova J, Plenio MB (2017). Protected ultrastrong coupling regime of the two-photon quantum Rabi model with trapped ions. Phys. Rev. A.

[3] Cheng XH (2018). Nonlinear quantum Rabi model in trapped ions. Phys. Rev. A.

[4] Felicetti S (2017). Two-photon quantum Rabi model with superconducting circuits. Phys. Rev. A.

[5] Brune M (1987). Realization of a two-photon maser oscillator. Phys. Rev. Lett..

[6] Bertet P (2002). Generating and probing a two-photon Fock state with a single atom in a cavity. Phys. Rev. Lett..

[7] Stufler S (2006). Two-photon Rabi oscillations in a single $$ In_{x}Ga_{1-x}As/GaAs$$ quantum dot. Phys. Rev. B.

[8] Del Valle E (2010). Two-photon lasing by a single quantum dot in a high-Q microcavity. Phys. Rev. B.

[9] Verma JK, Pathak PK (2016). Highly efficient two-photon generation from a coherently pumped quantum dot embedded in a microcavity. Phys. Rev. B.

[10] Qian C (2018). Two-photon Rabi splitting in a coupled system of a nanocavity and exciton complexes. Phys. Rev. Lett..

[11] Felicetti S, Hwang MJ, Boité AL (2018). Ultrastrong coupling regime of non-dipolar light-matter interactions. Phys. Rev. A.

[12] Ng KM, Lo CF, Liu KL (1999). Exact eigenstates of the two-photon Jaynes-Cummings model with the counter-rotating term. Eur. Phys. J. D.

[13] Ng, K.M., Lo, C.F. & Liu, K.L. Exact dynamics of the multiphoton Jaynes-Cummings model without the rotating-wave approximation. *Proceedings of the International Conference on Frontiers in Quantum Physics * (*July 9–11, 1997*) (eds Lim, S. C. *et al.*) 291–297 (Springer, 1998).

[14] Emary C, Bishop RF (2002). Exact isolated solutions for the two-photon quantum Rabi model. J. Phys. A Math. Gen..

[15] Travěnec I (2012). Solvability of the two-photon Rabi Hamiltonian. Phys. Rev. A.

[16] Maciejewski AJ, Przybylska M, Stachowiak T (2015). Comment on “Solvability of the two-photon Rabi Hamiltonian”. Phys. Rev. A.

[17] Travěnec I (2015). Reply to Comment on “Solvability of the two-photon Rabi Hamiltonian”. Phys. Rev. A.

[18] Duan L, Xie YF, Braak D, Chen QH (2016). Two-photon Rabi model: Analytic solutions and spectral collapse. J. Phys. A Math. Theor..

[19] Lupo E (2019). A continued fraction based approach for the two-photon quantum Rabi model. Sci. Rep..

[20] Cong L (2019). Polaron picture of the two-photon quantum Rabi model. Phys. Rev. A.

[21] Hu X (2019). The phase transition in two-photon Rabi model under mean field approximation. Int. J. Theor. Phys..

[22] Yan Z, Yao X (2020). Analytic solutions of two-photon Rabi model based on Bargmann space. IOP Conf. Ser. Mater. Sci. Eng..

[23] Armenta Rico RJ, Maldonado-Villamizar FH, Rodriguez-Lara BM (2020). Spectral collapse in the two-photon quantum Rabi model. Phys. Rev. A.

[24] Lo CF (2020). Demystifying the spectral collapse in two-photon Rabi model. Sci. Rep..

[25] Chen QH, Wang C, He S, Liu T, Wang KL (2012). Exact solvability of the quantum Rabi model using Bogoliubov operators. Phys. Rev. A.

[26] Cui S, Cao J, Fan H, Amico L (2017). Exact analysis of the spectral peroperties of the anisotropic two-bosons Rabi model. J. Phys. A Math. Theor..

[27] Von Roos O (1983). Position-dependent effective masses in semiconductor theory. Phys. Rev. B.

[28] Dekar L, Chetouani L, Hammann TF (1999). Wave function for smooth potential and mass step. Phys. Rev. A.

[29] Lo CF (2020). Manipulating the spectral collapse in two-photon Rabi model. Sci. Rep..

